# Editorial: Education in synthetic biology

**DOI:** 10.3389/fbioe.2026.1990539

**Published:** 2026-09-23

**Authors:** Pasquale Stano, Brian P. Ingalls, Si-Yu Li

**Affiliations:** 1 Department of Biological and Environmental Sciences and Technologies (DiSTeBA), University of Salento, Lecce, Italy; 2 Department of Applied Mathematics, University of Waterloo, Waterloo, ON, Canada; 3 Department of Chemical Engineering, National Chung Hsing University, Taichung, Taiwan

**Keywords:** bioengineering, biotechnology, education, learning, modeling, research training, synthetic biology, teaching methods

Synthetic Biology (SB) has emerged as a highly innovative field over the last 2 decades, bringing with it a vast array of tools, objectives, and significant recognition within the broader scientific community. As the field has grown, it has attracted researchers from a wide range of disciplines, including biology, bioengineering, chemistry, and materials science, resulting in a vibrant yet often fragmented landscape in which practitioners follow highly diverse educational and professional pathways. This diversity is characteristic of a young research area that addresses complex challenges but still lacks the formal and structured educational frameworks found in more established disciplines. Consequently, there is an urgent need to advance synthetic biology education by integrating these diverse bodies of knowledge and cultural perspectives to fully harness the field’s potential.

We launched this Research Topic (RT) to gather experiences and contributions related to education in SB, while also highlighting current research, educational approaches, and resources for students and educators from different backgrounds. We designed the RT to be broad, exploring the theoretical foundations and core scientific concepts required to effectively study, model, and engineer biological systems. In particular, we emphasized the role of interdisciplinary and transdisciplinary thinking, which represents a major conceptual and practical driver for the development of SB technologies in the years ahead. It is often repeated that, in the new century, biology and biotechnology will be central disciplines, and SB is their most modern implementation. However, responding to current challenges requires, on the one hand, highly technical specialization and, on the other hand, the capacity to think in terms of interconnected systems and to communicate effectively with other scientific (and non scientific) fields.

The nine articles included in this RT come from researchers directly involved in SB education and from those who are promoting modeling or applications. Here, we have clustered the contributions into three distinct subtopics to guide interested readers. These range from the delivery systems of education, to laboratory protocols, to student personal development.

To the first group, which can be identified with the process of delivering instructional components and the organization of synthetic biology education, we assign four papers. Veloz and Jendreiko have provided a foundational contribution by introducing Chemical Organization Theory (COT), a conceptual tool that formalizes SB concepts using reaction networks to teach systems thinking (Veloz and Jendreiko). By applying this formalism across various biological scales (from genetic circuits and metabolic engineering to synthetic consortia) the authors demonstrate how abstract concepts like emergence and resilience become computable and operational through the pyCOT platform. Their research indicates that students develop stronger systems thinking when they are introduced to these organizational concepts in familiar, non-biological systems before transferring them to biology. Another paper, contributed by Boada et al., details the successful integration of mathematical modeling into undergraduate curricula through a series of webinars and a comprehensive syllabus (Boada et al.). Originally developed to maintain student engagement during the COVID-19 pandemic, when laboratory access was restricted, these webinars and their accompanying scripts and case studies have proven to be an effective educational tool for both online and onsite learning environments. Complementing these specific pedagogical tools is a comprehensive and informative review, written by Menard et al. that stratifies synthetic biology education initiatives into classroom activities, course designs, and program-level curricula (Menard et al.). This review identifies growing opportunities in the development of low-cost education kits and provides a high-level guide for educators looking to convert traditional didactic courses into project-based learning environments through iterative design-based research. Finally, George et al. close this first group of papers by presenting a perspective about an engineering worldview of the field, specifically obtained through the lens of iGEM’s webinar series (George et al.). This series functions as a lasting, globally accessible blueprint for teaching technical knowledge and communication skills, aimed at reducing the barriers students face when attempting to enter this cutting-edge discipline.

The second cluster shifts the focus toward experimental protocols and technical applications, emphasizing hands-on methodology and the application of synthetic biology to real-world medical challenges. The first contribution, authored by Wang et al., explores the use of viral vector-based systems as a practical means of providing undergraduate students with hands-on research opportunities (Wang et al.). The authors highlight how these rapid, low-cost transient expression platforms can support authentic research experiences within a single academic term, while expanding an interdisciplinary access to SB, plant biotechnology, and STEM at primarily undergraduate institutions. This focus on the lab is further expanded by Morra et al. and his group in an article that describes a non-hypothesis-driven practical activity in functional metagenomics that teaches students how to “fish” for protein-coding DNA sequences from various microbiomes (Morra et al.). This approach allows biotechnology students to construct and screen metagenomic libraries from any intron-less genome, providing them with essential evidential knowledge of gene function that complements traditional sequence-based analysis. Another contribution in this cluster examines the transition from student competitions to clinical applications, specifically reviewing the development of live biotherapeutic products (LBPs) for anticancer therapy (Van den Berghe et al.). By analyzing 77 projects from the iGEM competition, the authors highlight how students are tackling the hallmarks of cancer–such as proliferation and metastasis–and leveraging the microbiome to innovate new diagnostic and therapeutic tools.

The remaining articles are mainly centered on personal development, examining how educational interventions impact the personal and professional identity of students within the scientific community, preparing them for future work in scientific contexts. This research is vital for strengthening the STEM pipeline and addressing retention issues, particularly for individuals from underrepresented groups. Mims et al. investigated the impact of the “BioBuilderClub”, a project-based intervention that significantly improved science identity, self-beliefs, and content knowledge among high school students across gender, location, and first-generation status (Mims et al.). The results demonstrate that engaging students in hands-on, interdisciplinary projects increases their interest in biotechnology and related fields. Finally, an article by Roberts and Farny illustrates that fostering student authorship and scientific writing skills, specifically regarding peer review and publishing, is essential for developing a scholarly identity (Roberts and Farny). The authors describe a project-based approach through which students gain expertise in specific application areas of SB. By measuring gains in scientific literacy and professional socialization, the study shows that these practices help students see themselves as active contributors to the research enterprise, thereby contributing to a more diverse and persistent field.

In conclusion, the RT articles emphasize that SB is not only a technological and scientific frontier, but also a rapidly evolving discipline whose future depends on effective and inclusive educational practices. By addressing key challenges such as the need for robust theoretical foundations, meaningful hands-on experiences, interdisciplinary integration, and personal development, the studies presented in this RT contribute to a broader reflection on how the next-generation of synthetic biologists should be trained. We hope that these contributions will help educators and researchers from diverse backgrounds develop innovative and high-quality learning environments, thereby strengthening the growing interface between biology and engineering and fostering the responsible advancement of SB. By integrating the development of communication skills and scholarly identity, we argue that SB education will progress not only from a technical viewpoint but also with greater ethical and social responsibility.

